# The Binaural Masking-Level Difference of Mandarin Tone Detection and the Binaural Intelligibility-Level Difference of Mandarin Tone Recognition in the Presence of Speech-Spectrum Noise

**DOI:** 10.1371/journal.pone.0120977

**Published:** 2015-04-02

**Authors:** Cheng-Yu Ho, Pei-Chun Li, Yuan-Chuan Chiang, Shuenn-Tsong Young, Woei-Chyn Chu

**Affiliations:** 1 Department of Biomedical Engineering, School of Biomedical Science and Engineering, National Yang-Ming University, Taipei City, Taiwan, R.O.C; 2 Holistic Education Center, Mackay Medical College, New Taipei City, Taiwan, R.O.C; 3 Department of Audiology and Speech-Language Pathology, Mackay Medical College, New Taipei City, Taiwan, R.O.C; 4 Department of Speech and Hearing Disorders and Science, National Taipei University of Nursing and Health Sciences, Taipei City, Taiwan, R.O.C; The University of Science and Technology of China, CHINA

## Abstract

Binaural hearing involves using information relating to the differences between the signals that arrive at the two ears, and it can make it easier to detect and recognize signals in a noisy environment. This phenomenon of binaural hearing is quantified in laboratory studies as the binaural masking-level difference (BMLD). Mandarin is one of the most commonly used languages, but there are no publication values of BMLD or BILD based on Mandarin tones. Therefore, this study investigated the BMLD and BILD of Mandarin tones. The BMLDs of Mandarin tone detection were measured based on the detection threshold differences for the four tones of the voiced vowels /i/ (i.e., /i1/, /i2/, /i3/, and /i4/) and /u/ (i.e., /u1/, /u2/, /u3/, and /u4/) in the presence of speech-spectrum noise when presented interaurally in phase (S_0_N_0_) and interaurally in antiphase (S_π_N_0_). The BILDs of Mandarin tone recognition in speech-spectrum noise were determined as the differences in the target-to-masker ratio (TMR) required for 50% correct tone recognitions between the S_0_N_0_ and S_π_N_0_ conditions. The detection thresholds for the four tones of /i/ and /u/ differed significantly (*p*<0.001) between the S_0_N_0_ and S_π_N_0_ conditions. The average detection thresholds of Mandarin tones were all lower in the S_π_N_0_ condition than in the S_0_N_0_ condition, and the BMLDs ranged from 7.3 to 11.5 dB. The TMR for 50% correct Mandarin tone recognitions differed significantly (*p*<0.001) between the S_0_N_0_ and S_π_N_0_ conditions, at –13.4 and –18.0 dB, respectively, with a mean BILD of 4.6 dB. The study showed that the thresholds of Mandarin tone detection and recognition in the presence of speech-spectrum noise are improved when phase inversion is applied to the target speech. The average BILDs of Mandarin tones are smaller than the average BMLDs of Mandarin tones.

## Introduction

Binaural hearing involves using information relating to the differences between the signals that arrive at the two ears, and it can make it easier to detect and recognize signals in a noisy environment. This phenomenon of binaural hearing is quantified in laboratory studies as the binaural masking-level difference (BMLD) [1]. The BMLD refers to the difference in the just-audible test-tone level when the signal or masker stimulus provided to one of the ears is changed. There are many possible stimulus conditions involving supplying different combinations of signal and masker to the ears. The most common scenario is where the phases of the maskers are the same at the two ears while the phase of the signal is zero at one ear (S_0_N_0_) and inverted at the other (S_π_N_0_) [2,3]. Previous studies of the BMLDs of normal-hearing (NH) subjects for pure tones have shown that the mean detection threshold differences are functions of frequency; for example, Hirsh [4] found that the BMLDs of pure tones of 100, 200, 500, 1000, 2000, and 5000 Hz were 5.5, 13.6, 10.8, 7.6, 2.5, and 2.6 dB, respectively. This indicates that the BMLD effect is dominant at low frequencies, especially from 200 to 500 Hz. In addition to the BMLDs of pure tones, some studies of BMLDs have also used speech as the target signal [5]. For example, Levitt and Rabiner [6] found a mean BMLD of 12.8 dB for monosyllable words, while Wilson et al. [7] found a mean BMLD of 9.4 dB for spondaic words.

The binaural hearing affects not only the detection task in noise but also affects the recognition task in noise. The binaural intelligibility-level difference (BILD) is defined as the difference in the 50% intelligibility levels between two binaural conditions [6]. In the following text of this study, the BILD referred to the difference in the intensity levels that correspond to 50% intelligibility in the S_0_N_0_ and the S_π_N_0_ condition respectively. The BILDs have used monosyllable and spondaic words as stimulus. Levitt and Rabiner [6] found that the mean BILD of monosyllable words was 5.7 dB for NH subjects, while the mean BILD for spondaic words was found to be 6.7 dB in English [8] and 7.2 dB in Swedish [7]. The previous studies showed that using the same spondaic format in different languages may result in variations of the BMLD and BILD values. This study therefore assumed that the BMLDs and BILDs of speech may vary with the language material used. While these studies have yielded English and Swedish BMLDs and BILDs, there are no publication values of BMLD or BILD on Mandarin speech, and currently there is no BMLD or BILD based on Mandarin speech used in clinical evaluations. Therefore, this study aimed to investigate the BMLD and BILD of Mandarin tones.

Mandarin is a tonal language with one of four basic tones allotted per syllable [9]. Tone differences may occur in other situations, such as the tone sandhi. This study focused on the phonetic aspect that many monosyllabic Mandarin words have the same syllable with different tones that convey different lexical meanings [10,11]; for example, /ma1/ means “mother,” /ma2/ means “sesame,” /ma3/ means “horse,” and /ma4/ means “to reprove.” Previous studies have demonstrated the importance of the fundamental frequency (f_0_) to the perception of the Mandarin tone [12,13], and it has been shown that f_0_ ranges from 35 to 250 Hz [14,15] for male and female voices. Howie [15] reported that different Mandarin tones exhibit different variations of f_0_; for example, male voices have tone 1 with a flat-high f_0_ of about 150 Hz, tone 2 with f_0_ rising from 115 to 150 Hz, tone 3 with f_0_ falling and then rising, from 113 Hz down to 40 Hz and then up to 113 Hz, and tone 4 with f_0_ falling from 157 to 105 Hz. Previous studies have also shown that other acoustical cues contribute to the perception of Mandarin tones, such as the temporal envelope and the duration [11,12,16,17]. The fundamental frequency is more resistant to noise than the temporal envelope cues in the perception of a Mandarin tone [18].

Experimental measurements of BMLDs involve listening to a signal in the presence of a masking noise, and so this study hypothesized that the main cue for Mandarin tone detection in noise is based on the variations of f_0_. If the BMLD of Mandarin tones shares similar features with the BMLD of pure tones, the range of BMLDs for different Mandarin tones is expected to be similar to the range of BMLDs based on pure tones from 100 to 200 Hz, which is the frequency range of the variations of f_0_ for Mandarin tones. On the other hand, if the BMLD of Mandarin tones shares similar features with the BMLD of speech, the BMLDs of Mandarin tones are expected to be similar to BMLDs based on monosyllabic words.

In addition, studies of the BMLDs and BILDs of speech signals [6,7] have revealed that the BMLD of a detection task is normally larger than the BILD of a recognition task for the same speech signal. The same Mandarin speech materials are used in detection and recognition tasks, and so the BMLDs of Mandarin tones are expected to be larger than the BILDs.

The study used two experiments to investigate the BMLDs and BILDs of Mandarin tones detection and recognition in the presence of noise.

## Materials and Methods

### Ethics statement

The use of human subjects in this study was reviewed and approved by the Institutional Review Board of National Yang-Ming University, Taiwan (IRB No.: 1000063). All subjects provided written informed consents to participate, and they were compensated for their travel expenses.

### Subject selection criteria

All of the subjects had NH, as determined by pure-tone audiometric tests indicating that their hearing thresholds were better than 25 dB HL at all octave frequencies from 250 to 8000 Hz [19]. The subjects were native Mandarin speakers and had an education level above high school in Taiwan.

### Instrumentation

The experiments were conducted in a double-walled sound booth designed for audiometric testing. The speech signals and noise signal were prepared using a self-developed computer program written in LabVIEW software (National Instruments), and they were sent to an external sound card (Creative model Live! 24-bit), a stereo headphone amplifier (Hou Fen HA-611 MKIII), and a pair of headphones (beyerdynamic DT 990 PRO). The sound card, headphone amplifier, and headphones were calibrated using a 1000-Hz pure-tone signal to produce an output level of 70 dB SPL. A sound level meter (Quest Model 1900) was used for calibration.

### Materials and stimuli

#### Target speech and masker

The voiced vowels /i/ and /u/ were chosen as the two monosyllabic bases on which four Mandarin tonal patterns were superimposed to form the eight (two syllables × four tones) test stimuli. These two vowels were chosen (rather than others such as /a/) in order to meet both criteria: (1) to maintain maximum articulatory/acoustic contrasts [20] and (2) to be morphologically realizable with all four tonal variations in Mandarin Chinese. (Note that /a/ was excluded because it is morphologically realizable with tone 1 only.) The two vowels /i/ and /u/ when paired with each of the four tones correspond to true words with different meanings: for example, /i1/ means “one,” /i2/ means “mother’s sister,” /i3/ means “chair,” and /i4/ means “liquid”; while /u1/ means “house,” /u2/ means “nothing,” /u3/ means “to dance,” and /u4/ means “do not.” Also, a vowel was chosen rather than a consonant-vowel syllable structure for testing in order to avoid variances in the results associated with the presence of consonant components. The fundamental-frequency characteristics of the target speech are listed in Table 1.

**Table 1:** Acoustic characteristics of the target signals.

	f_0_ min (Hz)	f_0_ mean (Hz)	f_0_ max (Hz)	f_0_ start (Hz)	f_0_ mid (Hz)	f_0_ end (Hz)	duration (ms)
/i1/	115	117	123	123	118	121	511
/i2/	99	106	127	127	101	127	462
/i3/	88	95	109	104	88	109	495
/i4/	77	123	171	171	121	77	439
/u1/	123	126	130	128	125	130	398
/u2/	100	110	135	111	103	135	515
/u3/	99	110	142	117	100	142	474
/u4/	86	138	207	207	130	86	309

Data are mean, maximum (max), minimum (min), start point, middle (mid) point, end point, and duration of the fundamental frequency of the target speech for the four tones of /i/ and /u/.

Carhart et al. (1966) showed that the BMLD may vary with the maskers employed [21]. This study used speech-spectrum noise as a masker because current clinical applications of speech BMLD or other types of speech audiometric assessments involving maskers use this type of noise as a masker, and also the ANSI/ASA S3.6–2004 standard suggests using it as a masker for speech stimuli [19,22]. The sound pressure level of the output at the test frequencies must be within 5 dB of a spectrum shape that is constant from 100 to 1000 Hz, and decline at a rate of 12 dB per octave from 1000 to 6000 Hz. This study used speech /i/ and /u/ as the target signals, and their f_0_ ranges from around 100 to 200 Hz. The speech-spectrum noise could mask effectively at 100 to 200 Hz, so this study chose speech-spectrum noise as the masker.

Silence periods of equal duration were padded before and after the recorded target speech so as to extend it to 3 seconds. A 3-second-long speech-spectrum noise signal was used as the masker at a level of 70 dB SPL. Stimuli that each had a total duration of 3 seconds were made by combining the target speeches at different levels and the masker signal at a fixed level of 70 dB SPL.

#### Material recording

The target speech stimuli /i/ and /u/ were recorded previously and had been used in the study of Tsai et al. [23]. The materials were recorded by a male voice, because the interword and intertalker variability are lower for male voices than for female voices [24]. Each monosyllable was recorded repeatedly until three audiologists agreed that the vocal quality, accent, and pronunciation were satisfactory. The speech-spectrum noise used as the masker was obtained from the digital recording on the compact disc (CD) accompanying Musiek and Rintelmann’s book [25], and the long-term spectrum of this noise was the same as the long-term spectrum of the speech signal.

#### Level adjustment

The stimuli used in the tests of BMLDs and BILDs were presented through the headphones. They comprised mixtures of the target speech and masker at different target-to-masker ratios. The amplitude of each recorded target speech signal was adjusted to the same root-mean-squared amplitude as the 1000-Hz pure tone used for calibration [19].

### Experimental design

#### Experiment 1

The BMLDs of Mandarin tone detection in the presence of speech-spectrum noise were determined based on the threshold differences of Mandarin tone detection between the S_0_N_0_ and S_π_N_0_ conditions.

Experiment 1 involved 20 NH subjects (11 males and 9 females) with ages ranging from 23 to 38 years (28.5±4.0 years, mean±SD).

The test employed 16 stimuli [2 vowels × 4 tones × 2 phase conditions (S_0_N_0_ and S_π_N_0_)]. All stimuli were presented in a random sequence. The initial level of the target speech was 75 dB SPL. The detection thresholds were determined by a two-down, one-up, changing-step-size procedure [26,27]. The initial step size was 10 dB, and was reduced to 2 dB after three reversals. Subjects used a mouse to enter a response into the computer when they detected the tone of the target speech presented via the headphones. Subjects were allowed to take a 10-minute break whenever necessary. The average time spent on the test was approximately 30 minutes.

#### Experiment 2

In experiment 2, the BILDs of Mandarin tone recognition in the presence of speech-spectrum noise were determined as the differences in the target-to-masker ratio (TMR) required for 50% correct tone recognitions between the S_0_N_0_ and S_π_N_0_ conditions. Tone recognition was measured as the percentage of correct answers for each subject under five TMR conditions (i.e., –7 to—19 dB), and the TMR corresponding to a 50% correct performance was estimated by interpolation and recorded as the BILD for that subject.

Experiment 2 involved 20 NH subjects (9 males and 11 females) with ages ranging from 18 to 23 years (19.4±1.2 years).

Experiment 2 involved 12 types of listening condition, comprising in quiet and 5 TMRs (–7, –10, –13, –16, and—19 dB) for the S_0_N_0_ and S_π_N_0_ conditions. The tests involved a total of 96 stimuli sets [2 vowels × 4 tones × 2 phase conditions (S_0_N_0_ and S_π_N_0_) × 6 target-to-masker conditions], with all of the stimuli delivered randomly to each subject.

Before starting the recognition test, the subjects were trained by listening to all of the target signals in a quiet environment until they were familiar with the test signals. The experiment was conducted by using a four-alternative, forced-choice procedure displayed on the computer screen for each trial. The next task trial was not presented until after the subject had responded to the present trial. The four alternative responses were displayed at the four corners of the monitor, equally distanced from the center. The mouse target moved to the center after each response. The symbols and text on the responses were phonetic symbols and descriptions, respectively, for the four tones. The subjects were asked to choose the correct answer on the computer with a mouse. If the subjects could not recognize the tone of the target speech, they were encouraged to make their best guess. The subjects were permitted to take a break for 10 minutes whenever they felt tired during the testing trials. Each subject participated in two trials in order to increase the number of testing tasks. Both trials used 96 sets of stimulus in different random sequences, and there was a 10-minute break between the two trials. These trials took an average of approximately 30 minutes to complete.

## Results

### Experiment 1

**Fig 1:**
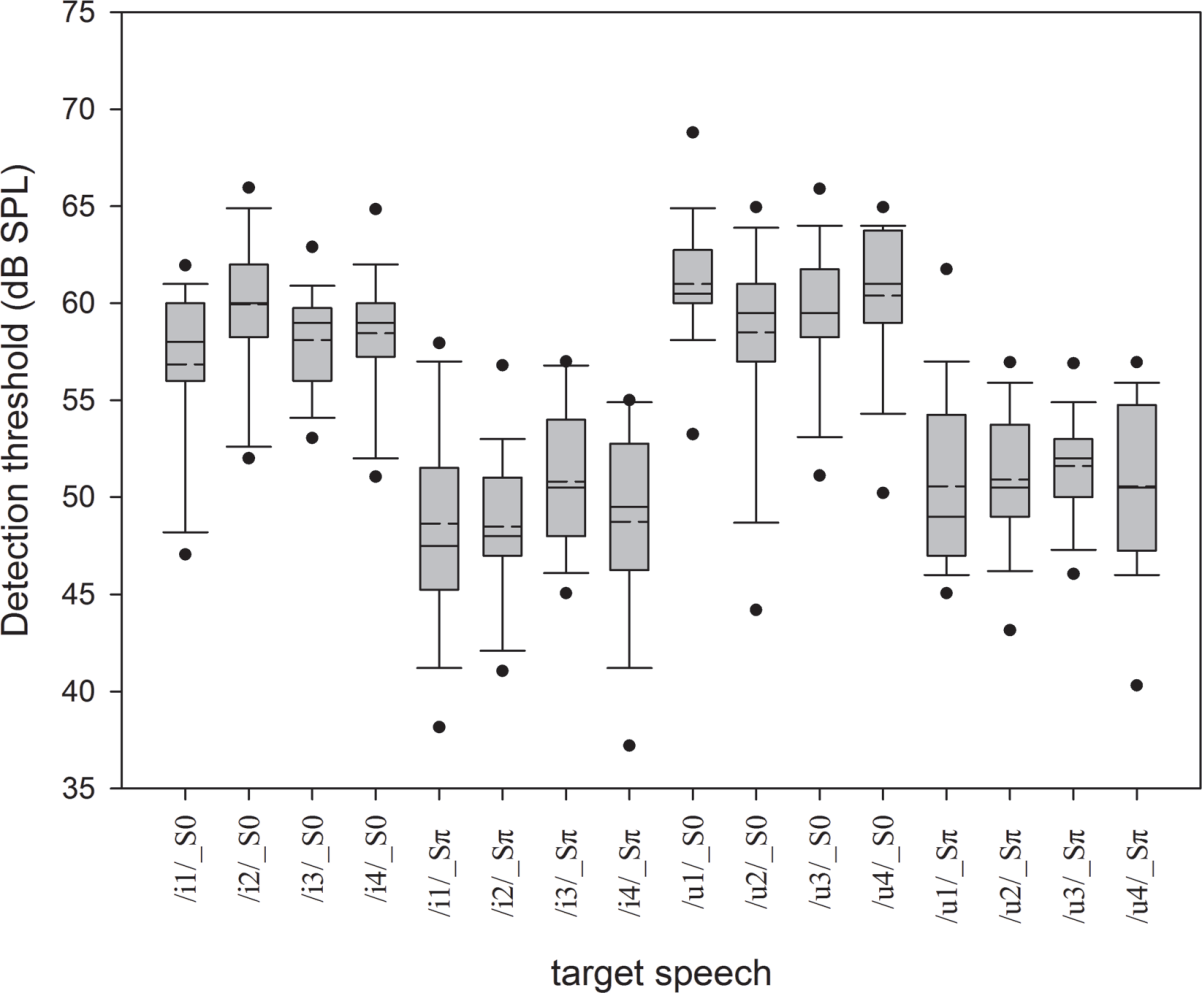
Average Mandarin tone detection thresholds in the S_0_N_0_ and S_π_N_0_ conditions. Each box plot shows the median (solid line), the mean (dash line), the 25^th^ and 75^th^ percentiles (box limits), the 10^th^ and 90^th^ percentiles (whiskers), and the 5^th^ and 95^th^ percentiles (black dots).

The average Mandarin tone detection thresholds in the S_0_N_0_ and S_π_N_0_ conditions for the four tones of /i/ and /u/ are shown in Fig 1 and tabulated in Table 2. The BMLDs derived from threshold differences (S_0_N_0_—S_π_N_0_) were 7.3 to 11.5 dB, and the mean BMLD of all of the target signals was 9.1 dB.

**Table 2:** The BMLDs of target speech (four tones of /i/ and /u/) for 20 NH subjects.

Target speech	mean of BMLD (dB)	SD	df	*p* value
/i1/	8.2	5.4	19	*p*<0.001
/i2/	11.5	4.0	19	*p*<0.001
/i3/	7.3	2.4	19	*p*<0.001
/i4/	9.7	3.4	19	*p*<0.001
/u1/	10.4	3.4	19	*p*<0.001
/u2/	7.6	4.3	19	*p*<0.001
/u3/	7.9	3.2	19	*p*<0.001
/u4/	9.8	4.0	19	*p*<0.001

Data are mean, standard deviation (SD), degree of freedom (df), and *p* values by paired-sample *t* test.

The paired-samples *t* test was used to evaluate whether the detection thresholds were better in the S_π_N_0_ condition than in the S_0_N_0_ condition. The results show that the detection thresholds differed significantly between the S_0_N_0_ and S_π_N_0_ conditions both for the four tones of /i/ [i.e., /i1/ (df = 19, *p*< 0.001), /i2/ (df = 19, *p*< 0.001), /i3/ (df = 19, *p*< 0.001), and /i4/ (df = 19, *p*< 0.001)] and for the four tones of /u/ [i.e., /u1/ (df = 19, *p*< 0.001), /u2/ (df = 19, *p*< 0.001), /u3/ (df = 19, *p*< 0.001), and /u4/ (df = 19, *p*< 0.001)]. In addition, the results in Table 2 indicate that all of the BMLDs of the tones differed from each other.

### Experiment 2

**Fig 2:**
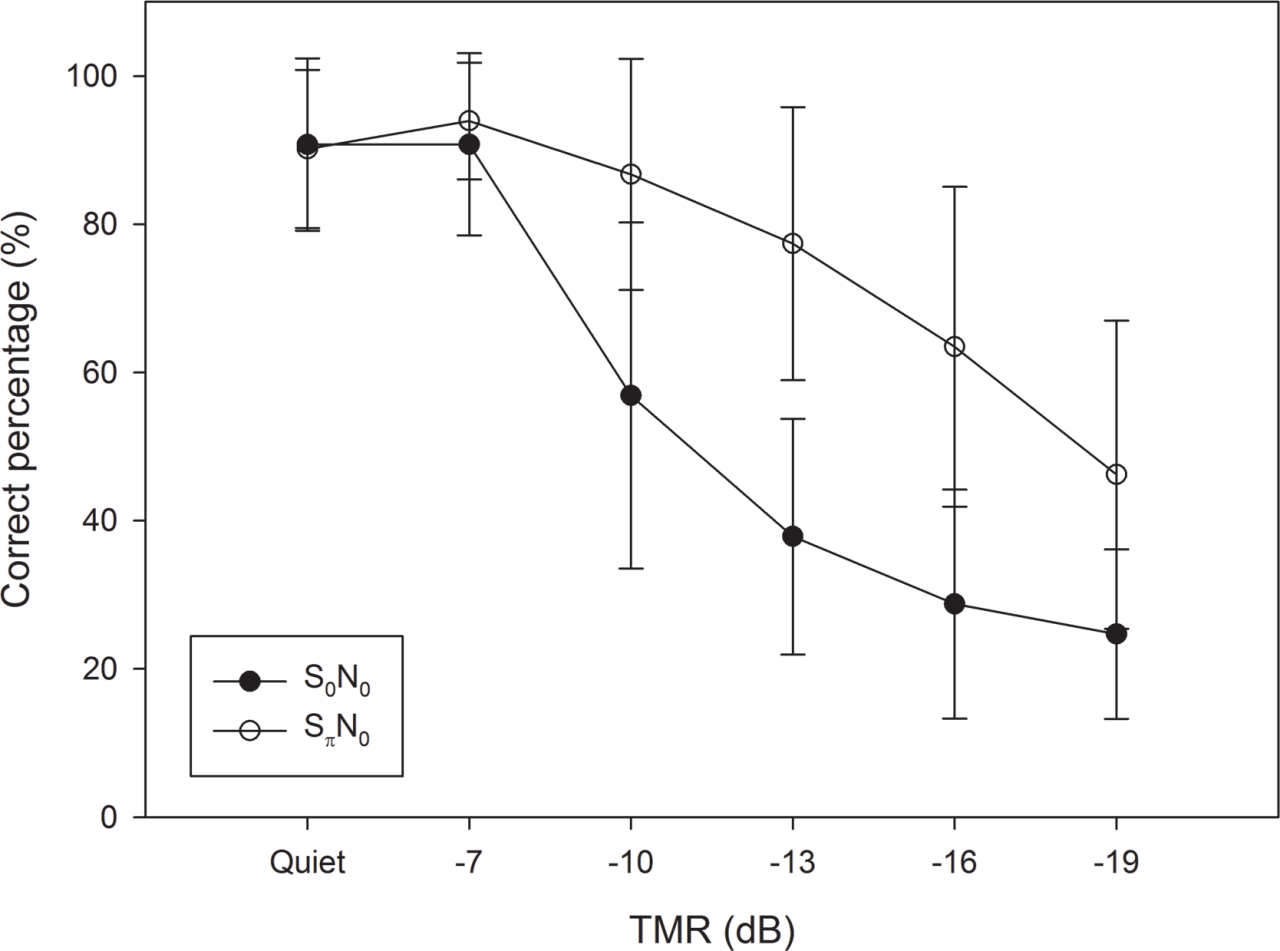
Mandarin tone recognition scores in quiet and TMR values in the S_0_N_0_ and S_π_N_0_ conditions. Data are mean and SD values.

The correct percentages of Mandarin tone recognition in quiet and for five TMRs in the S_0_N_0_ and S_π_N_0_ conditions are depicted in Fig 2, while the TMRs for 50% correct responses under these two conditions are tabulated in Table 3. The TMR was—13.4 dB for S_0_N_0_ and—18.0 dB for S_π_N_0_, and the mean BILD was 4.6 dB.

**Table 3:** The BILD of Mandarin tones recognition (four tones of /i/ and /u/).

mean of BILD (dB)	SD	df	*p* value
4.6	5.2	39	*p*<0.001

Data are mean, standard deviation (SD), degree of freedom (df), and *p* values from the paired-samples *t* tests.

The use of the paired-samples *t* test is to analyze whether the 50% of the correct responses corresponding to the TMR of Mandarin tone recognition were significantly improved in the S_π_N_0_ condition compared to the S_0_N_0_ condition, and the results revealed a significant difference (df = 39, *p* < 0.001). The paired-samples *t* test was also used to analyze whether each correct percentage of Mandarin tone recognition corresponding to each TMR was improved by phase inversion of the target signals. This revealed significant differences between the correct percentages of tone recognition in the S_0_N_0_ and S_π_N_0_ conditions for TMR values of—10 dB (df = 39, *p*< 0.001), –13 dB (df = 39, *p*< 0.001), –16 dB (df = 39, *p*< 0.001), and—19 dB (df = 39, *p*< 0.001), while there were no significant differences for quiet (df = 39, *p* = 0.700) and for TMR = –7 dB (df = 39, *p* = 0.099).

## Discussion

### Experiment 1

This study investigated the BMLDs of Mandarin tone detection and the BILDs of Mandarin tone recognition in the presence of speech-spectrum noise. The four tones of the voiced vowels /i/ (i.e., /i1/, /i2/, /i3/, and /i4/) and /u/ (i.e., /u1/, /u2/, /u3/, and /u4/) used in this study have fundamental frequencies ranging from 77 to 207 Hz. It is therefore reasonable to expect the BMLDs of these two sets of four tones to be within the range of the BMLDs of pure tones from 100 to 200 Hz. The mean BMLDs of Mandarin tone detection ranged from 7.3 to 11.5 dB, which are compatible with the range found for the BMLDs of pure tones (i.e., from 100 to 200 Hz; [4].)

The BMLDs differed for the four tones of /i/ and /u/ (Table 2). BMLD was 7.6 dB for /u2/ and 7.9 dB for /u3/; these values are very similar since /u2/ and /u3/ have similar f_0_ features in the speech material. The BMLD was largest for /i2/, but its f_0_ range is not the largest and its f_0_ value within this range is not the highest. There may be other factors affecting the BMLDs of Mandarin tone detection in noise, and more studies of the BMLDs of Mandarin speech are required to clarify these aspects.

The results showed the total mean BMLD was 9.1 dB for Mandarin tone detection, which contrasts with the mean BMLD of 12.8 dB found previously for English monosyllable words [6]. This is surprising given that the Mandarin tones were monosyllabic. The results of this study suggest that Mandarin tone detection in noise based on the variations of f_0_ may be analogous to pure-tone detection rather than to word detection. The results support the hypothesis that the main cue for Mandarin tone detection in noise depends on the variation of f_0_.

The Mandarin tone detection threshold differed significantly between the S_0_N_0_ and S_π_N_0_ conditions for all of the target signals used in this study (i.e., the four tones of /i/ and /u/). In other words, the Mandarin tone detection thresholds for these four tones of /i/ and /u/ are potentially improved by phase inversion of the target speech.

Based the criteria that used for material selection, this study chose two voiced vowels (/i/ and /u/) with four tones as the target signals. These vowels are produced by the advancement and height changes of the tongue [22], and they have similar f_0_ features but different formant features. Since this study wondered whether one of the vowels could represent another vowel, and whether the use of different vowels would affect the results of the BMLDs of Mandarin tone detection, this study applied interclass correlation (Pearson correlation) to the responses from the subjects between different vowels; that is, the BMLDs of /i1/ and /u1/. A statistical analysis of the interclass correlation produced the following results: /i1/ and /u1/ [r(i1, u1) = 0.114, r(i1, u1) denoting the Pearson correlation between /i1/ and /u1/, the sample size is 20], /i2/ and /u2/ [r(i2, u2) = 0.227], /i3/ and /u3/ [r(i3, u3) = 0.225], and /i4/ and /u4/ [r(i4, u4) = 0.556]. These results indicate that only the pair comprising /i4/ and /u4/ showed moderate correlation [28]; hence, except for tone 4, the use of different vowels would affect the BMLDs of Mandarin tones. The use of the four tones of /i/ and /u/ for BMLDs of Mandarin tones may not be sufficient to represent all of the tones in Mandarin speech. Furthermore, there may be other factors affecting the BMLDs of Mandarin tone detection in noise, such as differences in the formant distributions and the amplitude envelope. More studies of the BMLDs of Mandarin speech are needed to clarify such issues.

### Experiment 2

The measured BILDs of Mandarin tone recognition (Fig 2) revealed that the differences in the correct percentages between the S_0_N_0_ and S_π_N_0_ conditions increased for smaller TMR values. This is compatible with the results found in previous studies [7,8]. Although the mean TMR for 50% correct tone recognitions appeared to be improved when the condition changed from S_0_N_0_ to S_π_N_0_, the data need to be evaluated by a paired-samples *t* test to determine whether or not the differences are statistically significant. The statistical comparison of the 50% correct Mandarin tone recognitions between the S_0_N_0_ and S_π_N_0_ conditions revealed that the Mandarin tone recognition in the presence of speech-spectrum noise was improved by phase inversion of the target signals. The correct percentage of each tone recognition in noise for TMR values of—10, –13, –16, and—19 dB between the S_0_N_0_ and S_π_N_0_ conditions was improved by phase inversion of the target signals. In quiet, the speech signal presented at the two ears either in phase or out-of-phase sounded almost the same. As a result, the subjects performed almost the same in quiet and for TMR = –7dB, which suggests that the BILD will only be revealed in a noisy environment with a TMR lower than—10 dB. Moreover, a plateau may be reached depending on the characteristics of target and masker signals.

The mean BILD and BMLD values of the Mandarin tone recognition and detection tasks were 4.6 and 9.1 dB, respectively, in this study. The independent-samples *t* test was conducted to evaluate whether there is a significant difference between the BILD and BMLD values. The result showed that the variances of the two values exhibit no significant difference (df_1_ = 159, df_2_ = 39, *p* = 0.055), and the means of the BILD and BMLD are significantly different (df = 198, *p*<0.001). Previous studies found mean BMLDs of 12.8 and 9.4 dB, and mean BILDs of 5.7 and 7.2 dB for monosyllable and spondaic words, respectively. The results are therefore consistent with the trend that the BILDs are normally smaller than the BMLDs for the same speech signal [6,7].

The mean BILDs of tones 1–4 were 7.6, 2.5, 0.6, and 9.2 dB, respectively, as indicated in Table 4. The BILDs of tones 2 and 3 were notably lower than those of the other tones, which could have been due to the similarity of the f_0_ characteristics of the /u2/ and /u3/ speech material. Moreover, the correct percentages of tone 3 for S_0_N_0_ and S_π_N_0_ were 73.8% and 67.5% in the quiet condition, which might explain why /u2/ and /u3/ were easily confused in the speech material.

**Table 4:** Mean TMRs for 50% correct tone recognitions and mean BILDs between the S_0_N_0_ and S_π_N_0_ conditions for 20 NH subjects.

	Mean TMR for 50% correct recognitions (dB)		
	S_0_N_0_	S_π_N_0_	BILD (dB)
tone 1	–11.9	–19.5	7.6
tone 2	–12	–14.5	2.5
tone 3	–9	–9.6	0.6
tone 4	–11.2	–20.3	9.2

To investigate which tones are the main contributors to the correct percentages of tone recognition with phase inversion, confusion matrices of tone recognition were constructed using the results obtained for all of the testing conditions. Each cell in Table 5 presents the percentage of the particular response for the associated stimulus. The correct responses fall along the diagonal of the matrices, while the incorrect responses appear in the other cells; for example, cell SR_11_ corresponds to a stimulus of tone 1 and a response of tone 1, while cell SR_12_ corresponds to a stimulus of tone 1 and a response of tone 2.

**Table 5:** Confusion matrices of tone recognition in all testing conditions.

		S_0_N_0_				S_π_N_0_			
Condition		tone 1	tone 2	tone 3	tone 4	tone 1	tone 2	tone 3	tone 4
Quiet	tone 1	100	0	0	0	100	0	0	0
	tone 2	1.3	91.3	6.3	1.3	0	93.8	6.3	0
	tone 3	0	26.3	73.8	0	0	32.5	67.5	0
	tone 4	0	2.5	0	97.5	0	1.3	0	98.8
TMR (dB)		tone 1	tone 2	tone 3	tone 4	tone 1	tone 2	tone 3	tone 4
–7	tone 1	97.5	0	0	2.5	100	0	0	0
	tone 2	1.3	92.5	5	1.3	0	96.3	3.8	0
	tone 3	0	27.5	72.5	0	1.3	18.8	80	0
	tone 4	0	0	0	100	0	1.3	0	98.8
–10	tone 1	65	15	5	15	96.3**	2.5*	1.3	0*
	tone 2	10	65	16.3	8.8	3.8	90**	5*	1.3*
	tone 3	16.3	38.8	38.8	6.3	6.3*	22.5	66.3**	5
	tone 4	6.3	12.5	22.5	58.8	1.3	2.5*	2.5*	93.8**
–13	tone 1	41.3	22.5	30	6.3	87.5**	5*	1.3**	6.3
	tone 2	28.8	42.5	18.8	10	3.75**	81.3**	10	5
	tone 3	22.5	31.3	31.3	15	5*	33.8	47.5*	13.8
	tone 4	16.3	32.5	15	36.3	2.5*	0**	3.8*	93.8**
–16	tone 1	35	22.5	21.3	21.3	78.8**	7.5*	12.5	1.25**
	tone 2	12.5	48.8	17.5	21.3	12.5	60	16.3	11.3
	tone 3	38.8	35	13.8	12.5	17.5*	30	35*	17.5
	tone 4	23.8	30	28.8	17.5	10*	5**	2.5**	82.5**
–19	tone 1	26.3	35	22.5	16.3	53.8**	7.5**	22.5	16.3
	tone 2	32.5	30	27.5	10	22.5	40	27.5	10
	tone 3	35	25	26.3	13.8	17.5*	36.3	30	16.3
	tone 4	31.3	32.5	20	16.3	10*	12.5*	17.5	60**

One (*) or two (**) asterisks in the cells in the right-hand matrices indicate significant differences at *p*<0.05 or *p*<0.001, respectively, as evaluated by a paired-samples *t* test between the S_0_N_0_ and S_π_N_0_ conditions.

Under the testing conditions of quiet and TMR = –7 dB, the average correct percentages did not differ significantly between the S_0_N_0_ and S_π_N_0_ conditions, as evaluated by paired-samples *t* tests. The results also allow investigation of the overall tone recognition; the tendency was for the subjects to exhibit almost the same performance in quiet and for TMR = –7 dB. The paired-samples *t* test was then applied to each cell for TMR values of—10, –13, –16, and—19 dB to analyze whether the correct percentages in each cell differed significantly between the S_0_N_0_ and S_π_N_0_ conditions. For TMR = –10 dB the average correct percentage of each tone increased, and the average incorrect responses of SR_12_, SR_14_, SR_23_, SR_24_, SR_31_, SR_42_, and SR_43_ decreased with phase inversion of the target signal. For TMR = –13 dB the average correct responses of tones 1–4 increased, and the average incorrect responses of SR_12_, SR_13_, SR_21_, SR_31_, SR_41_, SR_42_, and SR_43_ decreased with phase inversion. For TMR = –16 dB the average correct responses of tone 1, tone 3, and tone 4 increased, and the average incorrect responses of SR_12_, SR_14_, SR_31_, SR_41_, SR_42_, and SR_43_ decreased with phase inversion. For TMR = –19 dB the average correct percentage was approximately 25%, which could be achieved by simply guessing the responses to four-alternative forced-choice questions. However, for TMR = –19 dB in the S_π_N_0_ listening condition, the average correct percentages exceeded 50% for tones 1 and 4. Therefore, in the TMR = –19 dB condition, the average correct percentage improved for tones 1 and 4 with phase inversion. This might also have been due to the /u2/ and /u3/ speech materials being too similar, resulting in confusion. In addition, the results of confusion matrices indicated that, on average, tones 1 and 4 were improved more with phase inversion from TMR = –10 dB to TMR = –19 dB. The f_0_ contours of tone 1 and 4 are more distinguishable than those of tones 2 and 3, and this may represent further evidence that the cue for tone recognition in noise is based on f_0_ contours.

In summary, all of the BMLDs of Mandarin tones obtained in this study agreed with the findings of previous studies. The BMLDs appear to be more affected by target signals with dominant frequencies ranging from 100 to 200 Hz. In particular, the BMLDs of Mandarin tone detection were in agreement with the range of BMLDs of 100- and 200-Hz pure tones. Furthermore, the detection and recognition of Mandarin tones in the presence of speech-spectrum noise are both improved in S_π_N_0_. This demonstrates the potential for improving Mandarin tones detection and recognition in noise.

Clinically, the BMLD has been suggested as an indicator of lesions that affect auditory pathways of the brainstem [29–31]. BMLD is measured using either a 500-Hz pure tone or speech [25]. In addition, Noffsinger et al. suggested that the BMLD for speech is more sensitive to auditory abnormalities in the brainstem than is the BMLD for pure tones. The BMLDs of the Mandarin vowels /i/ and /u/ with four tones may be applied to evaluate abnormalities in the auditory pathways of the brainstem in Mandarin speakers.

However, this study only used four tones of the vowels /i/ and /u/ as target speech signals, and hence the results for the BMLDs and BILDs of Mandarin tones should be viewed as being preliminary only. Further studies involving different vowels or consonant–vowel combinations of Mandarin tones are required to clarify the different effects of BMLDs and BILDs, with the aim of applying this information in clinical applications.

## Conclusion

This study has demonstrated that the average threshold for Mandarin tone detection in noise can be improved by inverting the phase of the target speech. Moreover, the range of BMLDs of the Mandarin tones agreed with the range of BMLDs of pure tones from 100 to 200 Hz. Based on the results, this study suggests that the f_0_ cue is the most robust one for Mandarin tone detection in noise.

In terms of the BILDs of Mandarin tone recognition, there were significant differences in the correct percentages between the S_0_N_0_ and S_π_N_0_ conditions for TMR = –10, –13, –16, and—19 dB, while in quiet and for TMR = –7 dB there are no significant differences between these two conditions.

The study has shown that the thresholds of Mandarin tone detection and recognition in the presence of speech-spectrum noise are improved when phase inversion is applied to target speech signals. Moreover, the average BILDs of Mandarin tones are smaller than the average BMLDs of Mandarin tones.
